# Ultrasound spatiotemporally enables prolonged therapeutic mRNA translation in engineered bacteria for enhanced cancer immunotherapy

**DOI:** 10.7150/thno.120342

**Published:** 2025-08-30

**Authors:** Zhaoyou Liu, Lantian Wang, Jieyuan An, Tian Zhou, Mengying Wei, Guodong Yang, Pengying Wu, Lijun Yuan

**Affiliations:** 1 Department of Ultrasound Medicine, Tangdu Hospital, Air Force Medical University, Xi'an, 710038, People's Republic of China.; 2 State Key Laboratory of Holistic Integrative Management of Gastrointestinal Cancers and Department of Biochemistry and Molecular Biology, Air Force Medical University, Xi'an 710032, People's Republic of China.

**Keywords:** bacteria-based therapy, cancer immunotherapy, genetic circuit, ultrasound, RNA thermometer

## Abstract

**Rationale:** Engineered bacteria have recently emerged as a novel and promising strategy for cancer immunotherapy. Nonetheless, precise spatiotemporal regulation of therapeutic gene expression within these bacteria is essential to optimize therapeutic efficacy while minimizing adverse effects. This study aims to develop a system for precise, ultrasound-driven regulation of gene expression in bacteria to enable targeted tumor therapy.

**Methods:** A modular system (Stabilized Open RNA thermometer, SORT) was designed, comprising a modified RNA thermometer with QKI response elements (QRE), therapeutic coding sequences, and the RNA binding motif of QKI. As a proof-of-concept, the bacteria VNP20009 was engineered with plasmids expressing mutated IL-2 or soluble PD-1 (sPD-1) within the SORT cassette. Syngeneic tumor mouse models (4T1 breast cancer and A20 lymphoma) were used to assess bacterial accumulation, therapeutic protein expression, anti-tumor immunity, and toxicity.

**Results:** Upon a single session of ultrasound irradiation, IL-2 or sPD-1 expression was efficiently and durably induced in the engineered VNP20009. In mouse tumor models, SORT-equipped VNP20009 accumulated in the tumor region and diminished from organs including the liver and lung. Ultrasound irradiation enabled the therapeutic protein (IL-2 or sPD-1) to be spatiotemporally switched-on within the tumor region. This localized expression resulted in robust activation of anti-tumor immunity alongside tolerable toxic effects.

**Conclusions:** The modular SORT platform provides a refined approach for bacteria-based therapy, enabling spatiotemporal control of therapeutic gene expression. This system enhances anti-tumor efficacy while reducing off-target toxicity, representing a promising strategy for cancer immunotherapy.

## Introduction

Engineered bacteria have demonstrated remarkable potential in treating a wide range of diseases 1,2, including gastrointestinal disorders 3,4, infectious diseases 5, metabolic disorders 6, and cancers 7-9. In cancer therapy, bacteria can specifically target tumors, actively penetrate tissues, and selectively colonize tumor sites 10-12. Advances in synthetic biology have further facilitated the design of bacteria capable of producing therapeutic agents in situ, which can either induce tumor cell apoptosis or modulate the tumor microenvironment (TME). Notably, these therapeutic agents are often harmful and thus toxic when bacteria are inevitably present in healthy tissues 13,14. To mitigate toxicity in healthy organs, spatiotemporal regulation of therapeutic gene expression in bacteria is critical.

To date, various genetic circuits have been developed to regulate therapeutic genes in bacteria 15. For example, the hypoxia response element (HRE), the promoter for NO detoxification gene norV (Pnorv) and arginine decarboxylase (adiA) promoter have been employed to control therapeutic genes expression under hypoxia 16, NO 17 and acidic tumor microenvironment respectively 18. External signals, such as isopropyl β-D-thiogalactoside (IPTG) 19, L-arabinose 20 and tetracycline 21 can be used to control the therapeutic gene expression driven by the lactose operon, pBAD promoter, and tetracycline-controlled transcriptional activator (tTA) or reverse tetracycline-controlled transcriptional activator (rtTA) respectively. Moreover, optogenetics 22 and sonogenetics 23 have also shown great potential in regulating bacterial gene expression. Compared with optogenetics, sonogenetics has attracted significant attention owing to its inherent advantages, such as noninvasiveness, high safety, and deep tissue penetration 24. Up to now, sonogenetics-mediated gene expression primarily relies on the ultrasound (US) thermal effect, and temperature-sensitive repressors (TSRs), such as TlpA and TcI, or promoters like leftward and rightward promoters (PL-PR), are usually used to control gene expression in response to US. For example, the repressor TlpA_39_ has been used to developed an US-responsive gene expression system, enabling efficient expression of Azurin or PD-L1 nanobody at 39 °C by US irradiation 25.

Additionally, RNA thermometers (RTs) can switch to an unfolded conformation in response to temperature increase and thus expose the ribosome binding site (RBS), allowing translation initiation 26. Theoretically, RTs could also be employed to develop genetic circuits activated by US.

**Figure 1:**
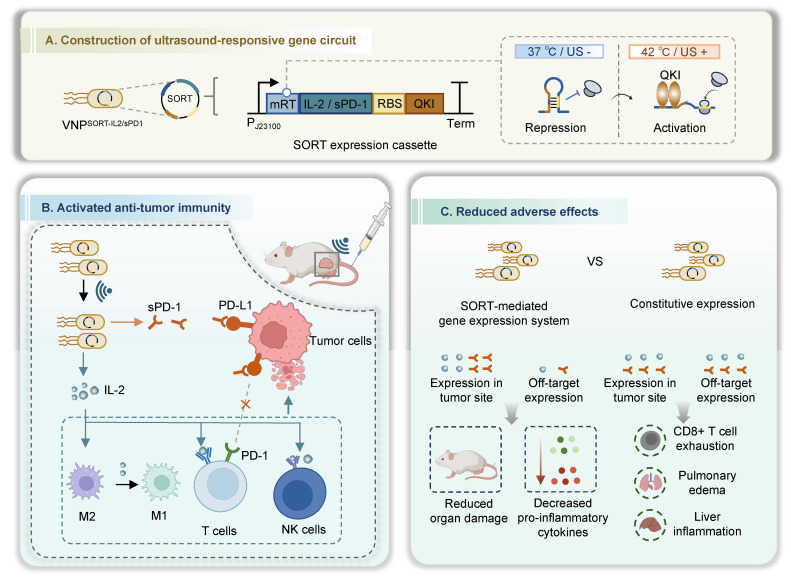
** Schematic illustration of ultrasound-triggered therapeutic mRNA translation in engineered bacteria for cancer immunotherapy. (A)** SORT-mediated gene expression system, in which the therapeutic mRNAs following the mRT could be translationally activated spatiotemporally by ultrasound.** (B)** Following intravenous administration, the bacteria target and colonize in the tumor sites. US induction switches IL-2 and/or sPD-1 expression in the tumor site, activating the anti-tumor immune response. **(C)** The SORT-mediated localized production and secretion of IL-2 or sPD-1 achieve robust therapeutic efficacy and minimized toxic effects. Figure created in Biorender.

Notably, to maintain the expression of therapeutic genes, continuous US irradiation is required to keep the temperature, which may be harmful and unsuitable in the context of treatments. Therefore, it is essential to develop a system capable to stably and durably express therapeutic gene following a brief US irradiation. In this study, we developed a stabilized open RNA thermometer (SORT) driven expression cassette, in which the RT was modified to harbor QKI response element (QRE), and thus could be opened by US irradiation and stabilized after QKI recognition (Figure 1). In the genetic circuit, the therapeutic open reading frame (ORF) following the RT could be translationally activated by US. As a proof-of-concept study, we engineered attenuated *Salmonella typhimurium* VNP20009 for US-triggered expression of mutated IL-2 or soluble PD-1 (sPD-1) using the genetic circuits. Our results demonstrated that US could spatiotemporally trigger production and secretion of IL-2 or sPD-1 in different syngeneic tumor mouse models, achieving robust therapeutic efficacy and minimizing toxic effects.

## Materials and Methods

### Plasmid construction and transfection

The IL-2, sPD1, and YopE peptide sequences were obtained from GenBank, and all plasmids constructed for this study (Table S1) were synthesized by GenScript (Nanjing, China). VNP20009 competent cells were prepared as described 20 and constructed plasmids were electroporated into these cells with a Gemini X2 system (BTX) at 2.5 kV. The engineered bacteria clones were subjected to sequence verification, and validated clones were stored at -80 °C for future use.

### Heat-induced mCherry expression in SORT system

The VNP^CRT-mCherry^ and VNP^SORT-mCherry^ were incubated at 37 °C or 42 °C for 10 min. Fluorescence microscopy (A1R, Nikon, Japan) was used to detect the fluorescence signal of bacteria. To optimize the SORT expression system, four RBS candidates, namely RBS1, RBS2, RBS3, and RBS4 were screened. The detailed RBS sequences were listed in Table S2, Supporting Information. The VNP^SORT-mCherry^ with different RBS candidates were incubated at 37 °C or 42 °C for 10 min, and the fluorescence signal of bacteria was detected under the fluorescence microscopy.

### US parameter optimization for SORT system

When VNP^CRT-mCherry^ and VNP^SORT-mCherry^ were cultured to an OD_600_ of 0.4-0.6, US induction was performed using an ultrasound experimental device (Hanil, Korea), with US coupling gel applied. Bacterial expression of mCherry was subsequently observed under fluorescence microscopy. US intensities of 1 W/cm^2^, 2 W/cm^2^, 2.5 W/cm^2^, and 3 W/cm^2^ were tested sequentially, and the temperature elevation of the culture medium was recorded. The duty cycle was adjusted to keep the temperature constant. In the *in vitro* experiments, US irradiation with optimized parameter (2 W/cm^2^, 1 MHz, 30% duty cycle) was used, which could keep the irradiated bacterium solution at 42 °C constant temperature.

For animal experiments, female BALB/c mice bearing tumors were anesthetized and subjected to US irradiation. The fiber optic probe was fixed at the tumor site for real-time recording of the temperature at the tumor site. Meanwhile, infrared imaging was employed to monitor the diffusion of ultrasonic thermal effects and the whole-body temperature of the mice. US intensities of 1 W/cm², 2 W/cm², 2.5 W/cm², and 3 W/cm² were explored, respectively. The duty cycle was adjusted to maintain a constant temperature. In the *in vivo* experiments, optimized parameter (2 W/cm^2^, 1 MHz, 30% duty cycle), which can keep the irradiated tumor tissue at a constant temperature of 42 °C, was used.

### RNA pull-down

The biotin-labeled oRT, mRT probes and linear RNA with QKI response element were synthesized by Tsingke (Beijing, China). Total protein of VNP^Con-QKI^ was extracted using the Bacterial Active Protein Extraction Reagent (Beyotime, China) according to the manufacturer's protocol. Biotin-labeled oRT and mRT probes were incubated at 37 °C or 42 °C for 10 min. Subsequently, 500 μg of total bacterial protein with RNase inhibitors was added to the probes, and the mixture was incubated with continuous rotation at 4 °C for 2 h. Streptavidin Magnetic Beads (Beyotime, China) were pre-equilibrated according to the manufacturer's protocol. The beads slurry (100 μL in Tris-buffered saline, TBS) was combined with the protein-RNA complexes, vigorously vortexed to ensure complete resuspension, and incubated at 4 °C for 16 h. Then, the beads were sequentially washed three times with 1× Washing Buffer. RNA-associated proteins were eluted using 1× SDS-PAGE Loading Buffer, followed by heat denaturation at 95 °C for 3 min. Magnetic separation was performed for 1 min, and the eluted proteins in the supernatant were subjected to Western blot analysis.

### Cell culture

4T1 cells were cultured in Roswell Park Memorial Institute (RPMI)-1640 medium with 10% fetal bovine serum and 1% penicillin-streptomycin. For the A20 cells, cells were grown in the same RPMI-1640 medium base, but with the additional inclusion of 0.01% 2-mercaptoethanol. Cells were grown at 37 °C in a 5% CO_2_ and were changed with fresh medium every other day.

### Animal housing and tumor inoculation

BALB/c female mice (8 weeks old) were purchased from the Lab Animal Center of the Air Force Medical University (Xi'an, China) and housed at a constant temperature on a 12 h light/dark cycle with five mice per cage and access to food and water. Animal experiments were approved by the Institutional Animal Care and Use Committee at the Air Force Medical University. About 1×10^6^ 4T1 cells were collected and suspended in 100 μL phosphate-buffered saline (PBS) prior to subcutaneous injection into the right breast fat pads of each mouse to establish 4T1 tumor models. For the construction of a distant tumor mouse model, 1×10^6^ 4T1 cells in 100 μL PBS were additionally injected into the left fat pads 3 days later. To establish the A20 tumor model, 1×10^6^ A20 cells in 100 μL RPMI without phenol red were injected subcutaneously. Tumor volumes were monitored every five days using vernier calipers and calculated according to the formula: Volume = a × b^2^/2, where a indicates the longer diameter and b indicates the shorter diameter.

### Bacterial *in vivo* distribution analysis

Ten days after tumor cell inoculation, 100 μL VNP^Con-mCherry^ at OD_600_ of 0.2 for each mouse was injected *via* the tail vein. To evaluate the biodistribution and tumor accumulation of bacteria in syngeneic tumor mouse model, the tumor-bearing mice were sacrificed and tumors and organs, including heart, lung, liver, kidney, and spleen, were collected for *ex vivo* fluorescence imaging by IVIS system (Thermo Fisher, USA) at the indicated times.

### US-triggered IL-2 expression

Ten days after tumor cell inoculation, 100 μL VNP^SORT-IL2^ at OD_600_ of 0.2 for each mouse was injected *via* the tail vein. On day 3 post-bacterial injection, tumor-bearing mice underwent ultrasound irradiation. Mice were euthanized 4 h later, and tumors alongside major organs were harvested. IL-2 expression at protein level was analyzed by western blot and ELISA. The expression of IL-2 mRNA was analyzed by Quantitative PCR (qPCR). The detailed primer sequences were listed in Table S3, Supporting Information.

### Flow cytometry

Tumor tissues were collected and cut into 1 mm^3^ pieces, which were transferred to DMEM medium containing 1 mg/mL collagenase IV (Gibco, USA) and digested for 40 min at 37 °C. The resulting suspensions were centrifuged at 800 × g for 5 min at 4 °C to obtain a cell pellet. The pellet was washed with Red Cell Lysis Solution (Biolegend, USA) to remove red blood cells. For analysis of the following surface markers, cells were stained with APC/Fire™ 750 anti-mouse CD45 (103154, BioLegend, USA), PerCP/Cyanine5.5 anti-mouse CD45 (103132, BioLegend, USA), FITC anti-mouse CD3 (100204, BioLegend, USA), PE/Cyanine7 anti-mouse CD8 (100722, BioLegend, USA), APC anti-mouse CD8 (100711, BioLegend, USA), PerCP/Cyanine5.5 anti-mouse CD4 (100540, BioLegend, USA), PE anti-mouse TIM3 (119703, BioLegend, USA), Brilliant Violet 421™ anti-mouse LAG-3 (125221, BioLegend, USA), PE anti-mouse CD49b (103506, BioLegend, USA), PE anti-mouse F4/80 (123110, BioLegend, USA) and APC anti-mouse CD80 (104714, BioLegend, USA), at 37 °C for 30 min. For CD206 staining, cells were fixed and permeabilized with Perm/Wash buffer (421002, BioLegend, USA) for 30 min according to the manufacturer's instructions following by incubation with APC anti-mouse CD206 (141708, BioLegend, USA). CytoFLEX (Beckman Coulter) or FACS Canto II (BD Biosciences) cytometers were used for the flow cytometry, and the data were analyzed using FlowJo V10 software.

### Toxicity analysis

Blood biochemistry analysis and hematoxylin and eosin (H&E) staining of heart, liver, spleen, lung, and kidney sections from the treated mice were used to evaluate the biosafety. For blood biochemistry analysis, blood samples were placed at room temperature for 2 h and then centrifuged at 350 × g for 30 min to obtain the blood serum. All biochemical serum evaluations were performed at the same time to minimize analytical variability. The degree of pulmonary edema was assessed by measuring the lung wet/dry weight ratio. The major organs were collected and fixed with 4 % paraformaldehyde overnight to perform H&E staining.

### Statistical analyses

All values were presented as mean ± SEM, and statistical significance was analyzed by GraphPad Prism 10.1.2 (GraphPad Software Inc, USA). The statistical tests used in this study included Student's t-test for comparing two independent groups and one-way analysis of variance (ANOVA) for multiple groups. Significant differences were considered at *P*<0.05.

## Results

### Design and characterization of SORT gene expression system

**Figure 2:**
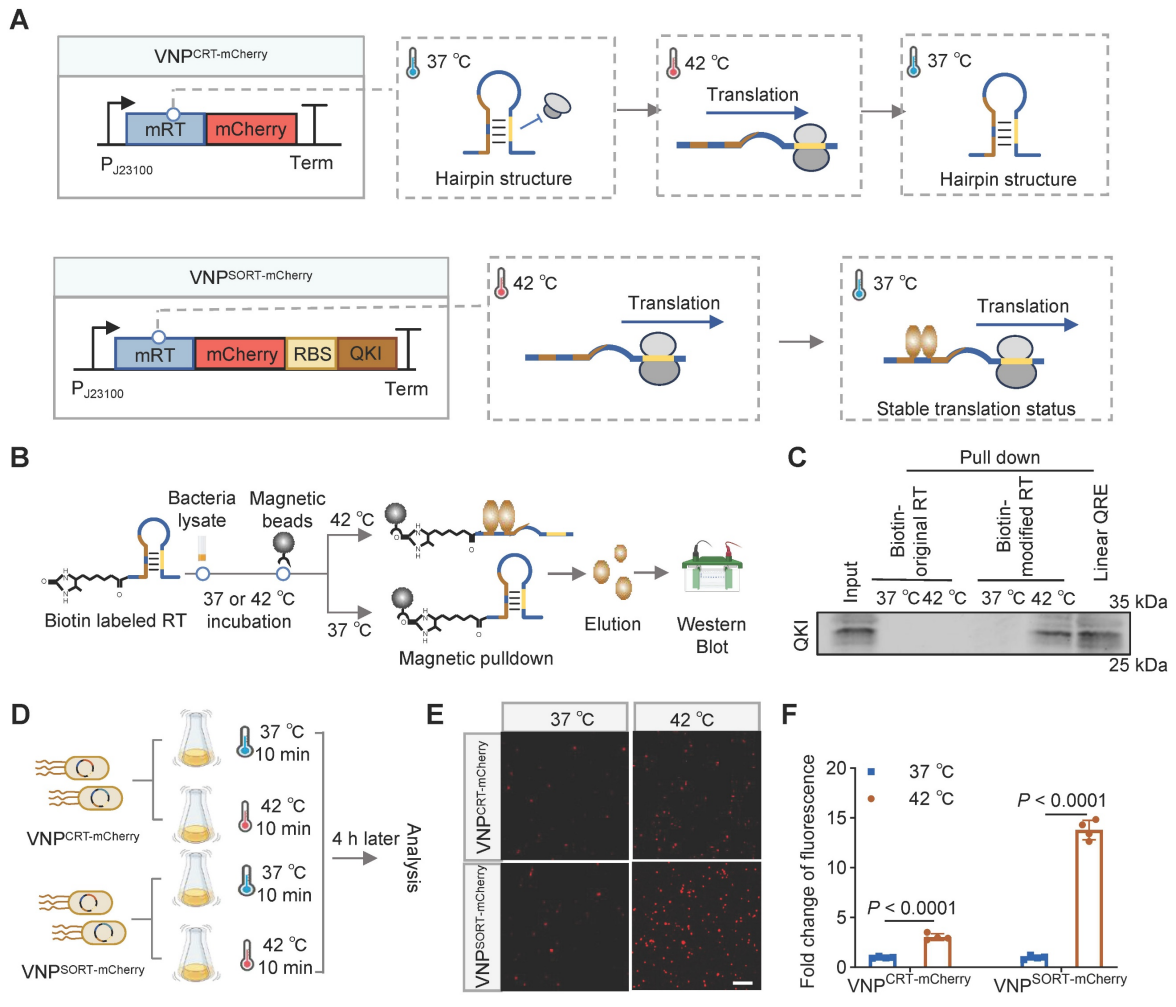
** Design and construction of SORT expression system. (A)** Schematic diagrams showing the structures and translation status of CRT-mCherry and SORT-mCherry during temperature switches. **(B)** Schematic of the experimental procedure of RNA pull-down assay. **(C)** Representative western blotting showing the QKI protein binds biotin-labeled mRT at 42 °C. **(D)** Schematic of the experimental procedure to assess heat-induced mCherry expression in VNP^CRT-mCherry^ and VNP^SORT-mCherry^. **(E)** Representative fluorescence microscopy images showing mCherry expression in VNP^CRT-mCherry^ and VNP^SORT-mCherry^ at 37 °C and 42 °C. (scale bar = 50 μm). **(F)** Fold change of fluorescence intensity in VNP^CRT-mCherry^ and VNP^SORT-mCherry^ at 37°C and 42 °C. Data are presented as mean ± SEM (n = 4 independent experiments). *p* values were calculated by Student's t-test.

The RT regulating the expression of inclusion body-binding protein A (IbpA) in *Pseudomonas putida* was employed to establish the US-responsive gene expression system 27. Mechanistically, the original RT (oRT) switches from a folded to an unfolded state when the temperature increases from 37 °C to 42 °C, thereby permitting the translation of IbpA downstream of the RT. In this study, oRT was modified (mRT) to contain two QREs within the hairpin structure, enabling recognition by the QKI protein (Figure S1). Theoretically, once mRT undergoes heat-induced conformational changes, it will be bound by the co-exist QKI protein, and prevented from turning back to the hairpin structure. The stable-open structure thus initiates a stable and durable therapeutic gene expression (Figure 2A). RNA pull-down assays demonstrated that QKI could bind mRT at 42 °C (Figure 2B-C). Hereafter, we named the mRT together with QKI as the SORT system, while the cassette without QKI served as the control (CRT). As expected, a transient thermal elevation (42 °C for 10 min) induced a 5.16-fold increase in mCherry expression in VNP^SORT-mCherry^ relative to VNP^CRT-mCherry^ (Figure 2D-F). In addition, either SORT-mCherry or CRT-mCherry engineering had no adverse effects on viability (Figure S2). Notably, the RBS affects the expression efficiency of QKI. Among four screened RBS variants screened, RBS2 displayed the best induction effect (Figure S3), and was thus selected for subsequent experiments.

**Figure 3:**
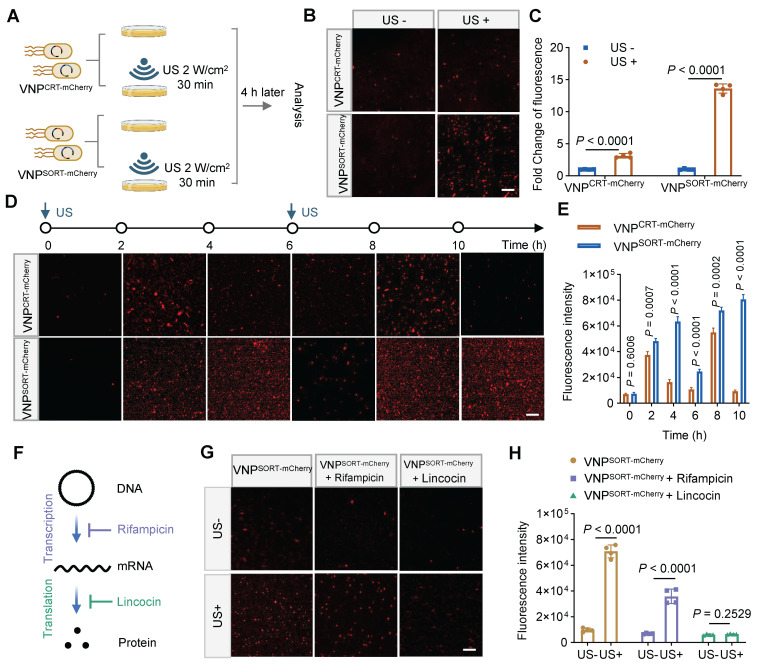
** Ultrasound stably triggers gene translation in SORT expression system. (A)** Schematic of the experimental procedure. US-induced mCherry expression in VNP^CRT-mCherry^ and VNP^SORT-mCherry^ following US irradiation was analyzed under microscopy. **(B)** Representative fluorescence microscopy images of VNP^CRT-mCherry^ and VNP^SORT-mCherry^ with and without US irradiation. (scale bar = 50 μm). **(C)** Fold change of fluorescence intensity of VNP^CRT-mCherry^ and VNP^SORT-mCherry^ with and without US irradiation. **(D)** Representative fluorescence microscopy images of VNP^CRT-mCherry^ and VNP^SORT-mCherry^ at different times after US irradiation. (scale bar = 50 μm). **(E)** Quantitative results of** (D)**. **(F)** The mechanisms how rifampicin and lincocin block gene expression. **(G)** Representative fluorescence microscopy images of the control group, the rifampicin group, and the lincocin group with and without US treatment. **(H)** Quantitative results of **(G)**. Data are presented as mean ± SEM (n = 4 independent experiments). *p* values were calculated by Student's t-test.

Subsequently, we investigated the performance of the SORT system under US irradiation. Among the parameters evaluated, US at 2 W/cm² with a 30% duty cycle elevated the bacterial culture temperature to approximately 42 °C (Figure S4). Consistent with thermal induction, mCherry fluorescence in VNP^SORT-mCherry^ was significantly increased under US induction (Figure 3A-C). In contrast, mCherry fluorescence in VNP^CRT-mCherry^ was markedly lower. Furthermore, mCherry protein production in VNP^SORT-mCherry^ was sustained for an extended period after the brief US irradiation (Figure 3D-E). Additionally, transcriptional inhibitor rifampicin 28 and translational inhibitor lincomycin 29 blocked US-triggered fluorescence expression in VNP^SORT-mCherry^, confirming that SORT-mediated regulation was at translational level (Figure 3F-H). The above results indicated that a brief session of US induction could stably and durably express the mCherry in the SORT system.

### Effective and specific translation of IL-2 in VNP^SORT-IL2^ triggered by US

**Figure 4:**
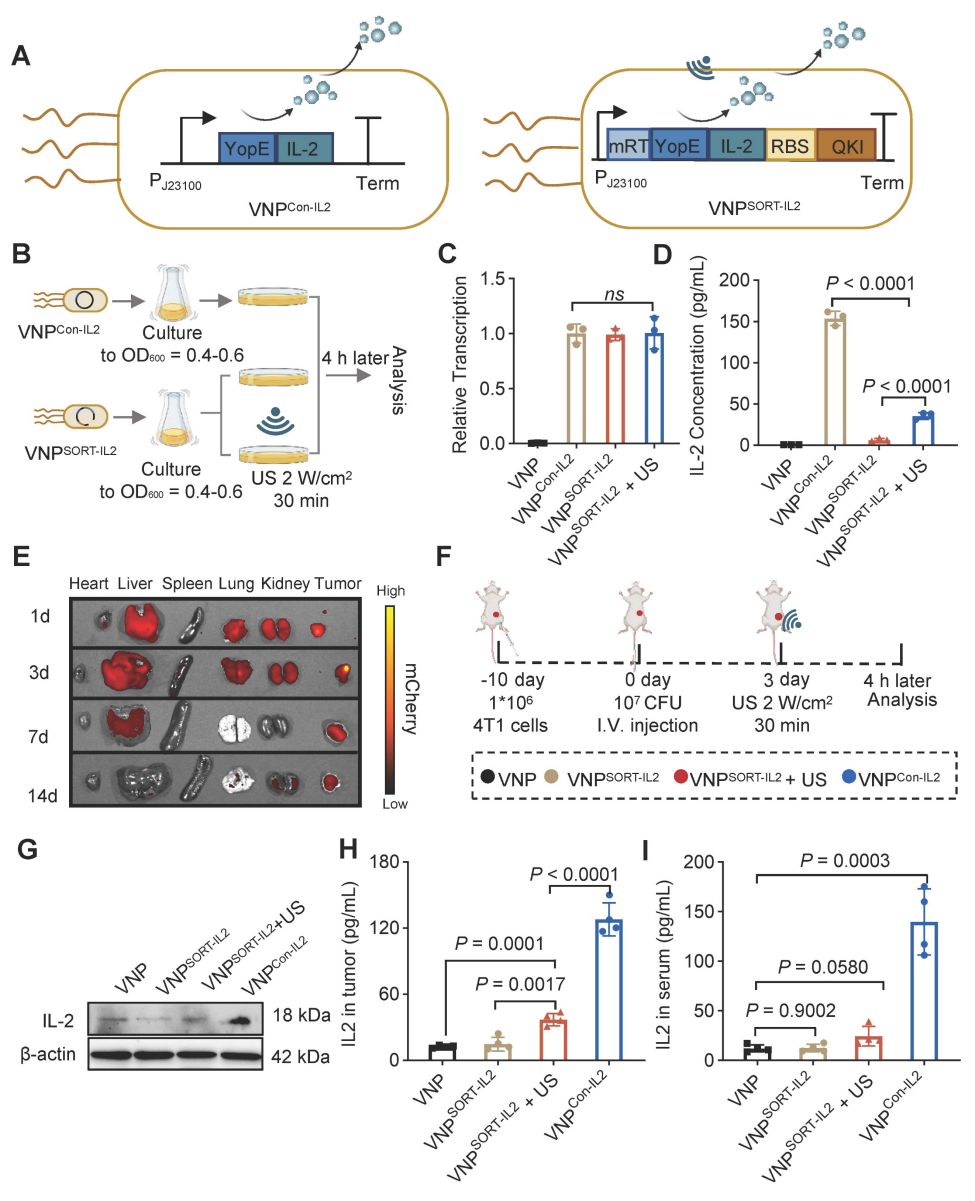
** Construction and characterization of VNP^SORT-IL2^. (A)** Schematic illustration of constitutive IL-2 expression and secretion in VNP^Con-IL2^ (Left) and US-triggered IL-2 expression and secretion in VNP^SORT-IL2^ (Right). **(B)** Schematic of the experimental procedure assessing the IL-2 expression in VNP^Con-IL2^ and VNP^SORT-IL2^ with or without US. **(C)** qPCR analysis of IL-2 mRNA expression. Data are presented as mean ± SEM (n = 3 independent experiments). **(D)** Quantitative analysis of IL-2 protein content in VNP^Con-IL2^ and VNP^SORT-IL2^ culture medium. Data are presented as mean ± SEM (n = 3 independent experiments). **(E)** Representative *ex vivo* fluorescent images of various organs and tumor tissues in mice, captured at days 1, 3, 7, 14 following tail vein injection of VNP^Con-mCherry^. **(F)** Schematic of the experimental procedure to assess the IL-2 expression in VNP-, VNP^SORT-IL2^-, and VNP^Con-IL2^-treated tumors. **(G)** Representative western blotting image of IL-2 expression in tumor tissues. **(H-I)** Quantitative analysis of IL-2 protein content in tumor **(H)** and serum **(I)** by ELISA. Data are presented as mean ± SEM (n = 4 mice per group). *p* values were calculated by one-way ANOVA.

IL-2 is a potent cytokine with multiple biological functions, including promoting T cell proliferation, differentiation, and effector activity, as well as enhancing natural killer (NK) cell activity 30. However, systemic delivery of IL-2 can cause severe toxicity, which limits its clinical application 31. SORT system was thus employed to enable spatiotemporal IL-2 expression under US induction. Briefly, VNP^SORT-IL2^ was engineered by replacing mCherry with YopE-IL-2 fusion gene (Figure 4A). VNP^Con-IL2^, which constitutively expresses and secretes IL-2, was constructed as a control (Figure 4A). VNP^SORT-IL2^ was subjected to US irradiation following the procedure described in Figure 4B. qPCR analysis confirmed no significant differences in IL-2 mRNA levels among VNP^Con-IL2^ and VNP^SORT-IL2^ with or without US irradiation (Figure 4C). In contrast, ELISA analysis of bacterial supernatants showed that US irradiation triggered IL-2 translation and secretion in VNP^SORT-IL2^, though the amount was still much less than that in VNP^Con-IL2^ (Figure 4D). Additionally, repeated US further enhanced IL-2 expression and secretion in VNP^SORT-IL2^ (Figure S5).

Having validated the *in vitro* functionality of the SORT system, we next evaluated the US-triggered translation efficiency of VNP^SORT-IL2^ in a 4T1 syngeneic mouse model. Consistent with previous study 32, VNP bacterial accumulation peaked in the liver within 1-day post-injection, and tumor-associated bacterial loads exceeded liver levels since day 3, with nearly complete clearance in non-target organs on day 14 (Figure 4E; Figure S6). Mice exhibited a baseline body temperature of 37-37.5 °C, and peaked up to 38.8 °C post-injection of VNP20009 (Figure S7). This temperature range remained well below the 42 °C activation threshold of mRT, thereby confirming the controllability and safety of the SORT system *in vivo*. To activate the SORT system *in vivo*, the suitable ultrasonic parameters including intensity and duty cycle, were explored. US irradiation on the tumor (2 W/cm², 30% duty cycle) elevated the temperature to a stable range of 42-43 °C (Figure S8).

VNP, VNP^Con-IL2^ and VNP^SORT-IL2^ were injected 10 days post-tumor inoculation in a 4T1 syngeneic mouse model. US was additionally applied to the tumor region in the VNP^SORT-IL2^ group 3 days later (Figure 4F). Western blot and ELISA confirmed that US triggered IL-2 expression and secretion specifically within the tumor (Figure 4G-H). Compared to VNP^Con-IL2^, VNP^SORT-IL2^ + US group exhibited lower IL-2 levels in serum and non-tumor tissues (Figure 4I; Figure S9). These results demonstrated efficient and spatially restricted expression and secretion of IL-2 in VNP^SORT-IL2^ induced by US.

### VNP^SORT-IL2^ combined with US inhibits tumor growth with reduced systemic toxicity

**Figure 5:**
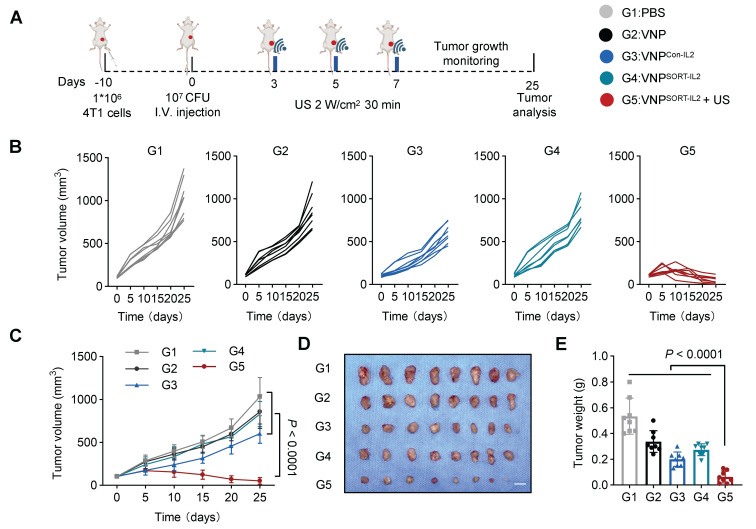
** Therapeutic effects of VNP^SORT-IL2^ in 4T1 syngeneic mouse model. (A)** Schematic of the experimental procedure. BALB/c mice bearing 4T1 breast tumors received treatments of PBS, VNP, VNP^Con-IL2^, VNP^SORT-IL2^, or VNP^SORT-IL2^ + US. **(B)** Individual growth curves of 4T1 breast tumors in different groups. **(C)** Statistical analysis of tumor growth in mice with different treatments. **(D)** Excised tumors from the mice with different treatments. **(E)** Tumor weights in different groups. Data are presented as mean ± SEM (n = 8 mice per group). *p* values were calculated by one-way ANOVA.

To evaluate the efficacy of SORT system-based therapy, 4T1 tumor-bearing mice were administered with PBS, VNP, VNP^Con-IL2^, or VNP^SORT-IL2^ *via* tail vein 10 days post-tumor inoculation. For VNP^SORT-IL2^ + US group, US irradiation (2 W/cm², 30% duty cycle, 30 min) was additionally applied on days 3, 5, and 7 post bacteria injection (Figure 5A). VNP^SORT-IL2^ + US group exhibited most significant tumor growth suppression, compared to other groups (Figure 5B-E). VNP and VNP^SORT-IL2^ without US also showed moderate tumor inhibition, which is likely attributable to the innate immune activation induced by bacteria.

**Figure 6:**
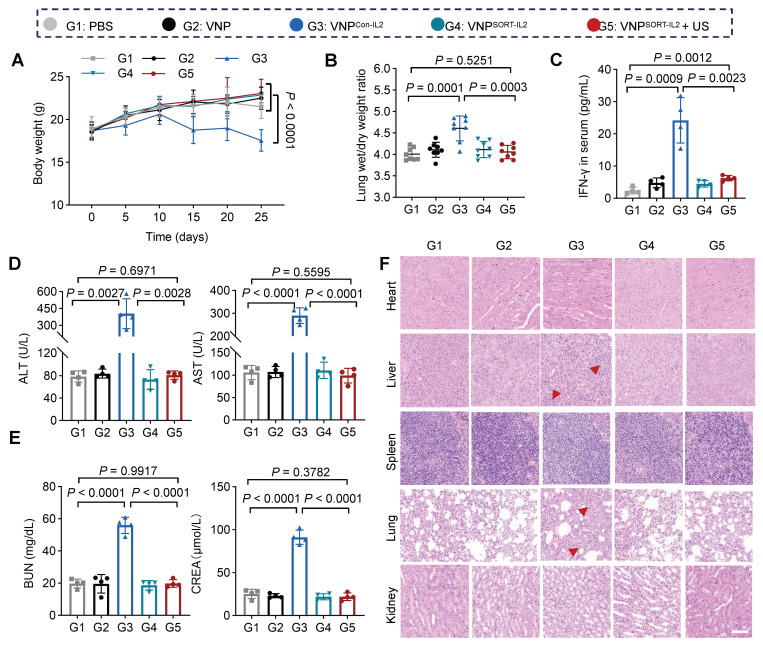
** Side-effects of VNP^SORT-IL2^. (A)** Body weights of the different groups. **(B)** Lung wet/dry weight ratio of 4T1 tumor-bearing mice in different groups. **(C)** Serum level of IFN-γ in different groups. **(D)** Serum levels of ALT and AST in different groups. **(E)** Serum levels of BUN and CREA in 4T1 tumor-bearing mice with different treatments. **(F)** Representative images of H&E staining of indicated organs from mice with different treatments. Scale bar = 100 μm. Red arrowheads indicate hepatic inflammation and pulmonary edema. Data are presented as mean ± SEM (n = 4-8 mice per group). *p* values were calculated by one-way ANOVA.

Although the tumor reduction was also remarkable for VNP^Con-IL2^, it caused severe adverse effects, including weight loss (Figure 6A), pulmonary edema (Figure 6B), as indicated by lung wet/dry weight ratios, serum interferon-γ (IFN-γ) levels (Figure 6C), biomarkers of hepatic and renal dysfunction (ALT, AST, BUN, CREA) (Figure 6D-E), as well as lymphocytic infiltration in hepatic and renal tissues (Figure 6F). In contrast, these side effects were merely observed in VNP^SORT-IL2^ + US group. Collectively, these findings demonstrated that VNP^SORT-IL2^ + US exhibited sound therapeutic activity with reduced systemic toxicity.

### VNP^SORT-IL2^ combined with US elicits CD8^+^ T cell activation

**Figure 7:**
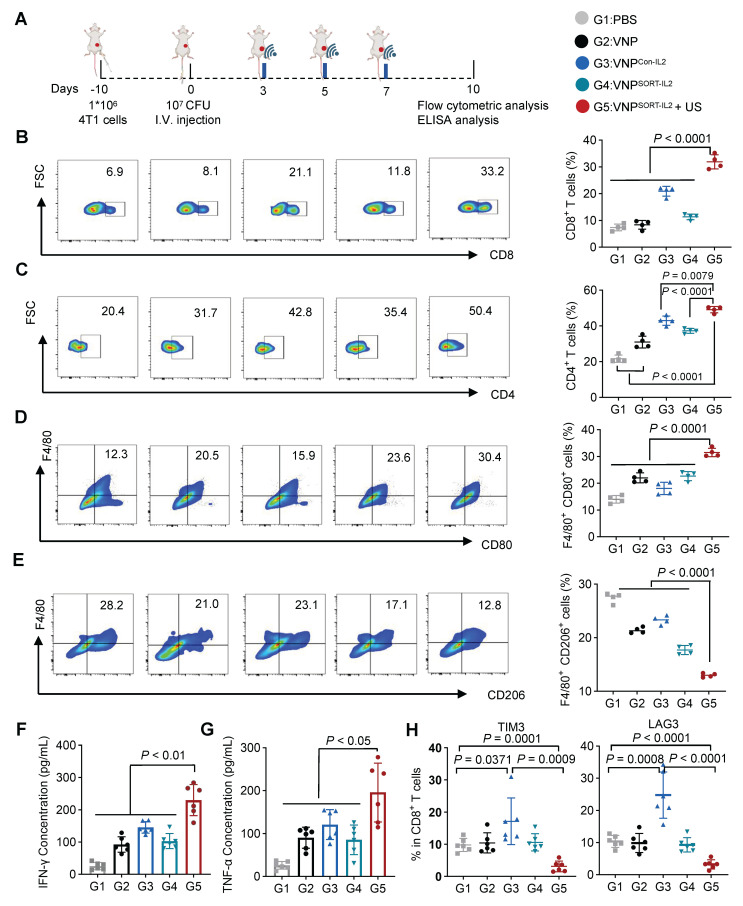
** VNP^SORT-IL2^ elicits immune activation. (A)** Schematic of the experimental procedure. BALB/c mice bearing 4T1 breast tumors received intravenous injections of PBS, VNP, VNP^Con-IL2^, VNP^SORT-IL2^, or VNP^SORT-IL2^ + US. **(B-E)** Flow cytometric analysis of CD8^+^ T cells (CD3^+^ CD8^+^) **(B)**, CD4^+^ T cells (CD3^+^ CD4^+^) **(C)**, M1 macrophages (F4/80^+^ CD80^+)^ **(D)** and M2 macrophages (F4/80^+^ CD206^+^) **(E)** in tumors of different treatments. **(F, G)** IFN-γ **(F)**, TNF-α **(G)** levels in tumors in different groups. **(H)** Flow cytometric quantification of TIM3^+^ and LAG3^+^ in CD8^+^T cells in the tumor. Data are presented as mean ± SEM (n = 4-6 mice per group). *p* values were calculated by one-way ANOVA.

To profile the immune response elicited by the VNP^SORT-IL2^ + US treatment, tumors and lymph nodes were harvested on day 10 post-bacteria administration and analyzed by flow cytometry (Figure 7A). VNP^SORT-IL2^ combined with US significantly enhanced antitumor immunity compared to other groups, as evidenced by elevated infiltration of T cells, particularly CD8+ (Figure 7B-C; Figure S10A-B), increased percentage of M1 macrophages (F4/80^+^ CD80^+^) (Figure 7D; Figure S10C), and decreased percentage of M2 macrophages (F4/80^+^ CD206^+^) (Figure 7E; Figure S10D). ELISA quantification of tumor lysates further revealed pronounced upregulation of IFN-γ (Figure 7F) and TNF-α (Figure 7G) in the VNP^SORT-IL2^ + US group. Although VNP^Con-IL2^ also promoted CD8^+^ T cell proliferation, it failed to sustain M1 polarization, which was consistent with its suboptimal tumor control 33. Moreover, there were higher amount of TIM3^+^ and LAG3^+^ CD8^+^ T cells in the VNP^Con-IL2^ group than that in VNP^SORT-IL2^ + US (Figure 7H). All the data suggested that the VNP^SORT-IL2^ + US strategy elicited T cell activation while avoiding T cell exhaustion.

### VNP^SORT-IL2^ combined with US suppresses the growth of distant/metastatic tumor

**Figure 8:**
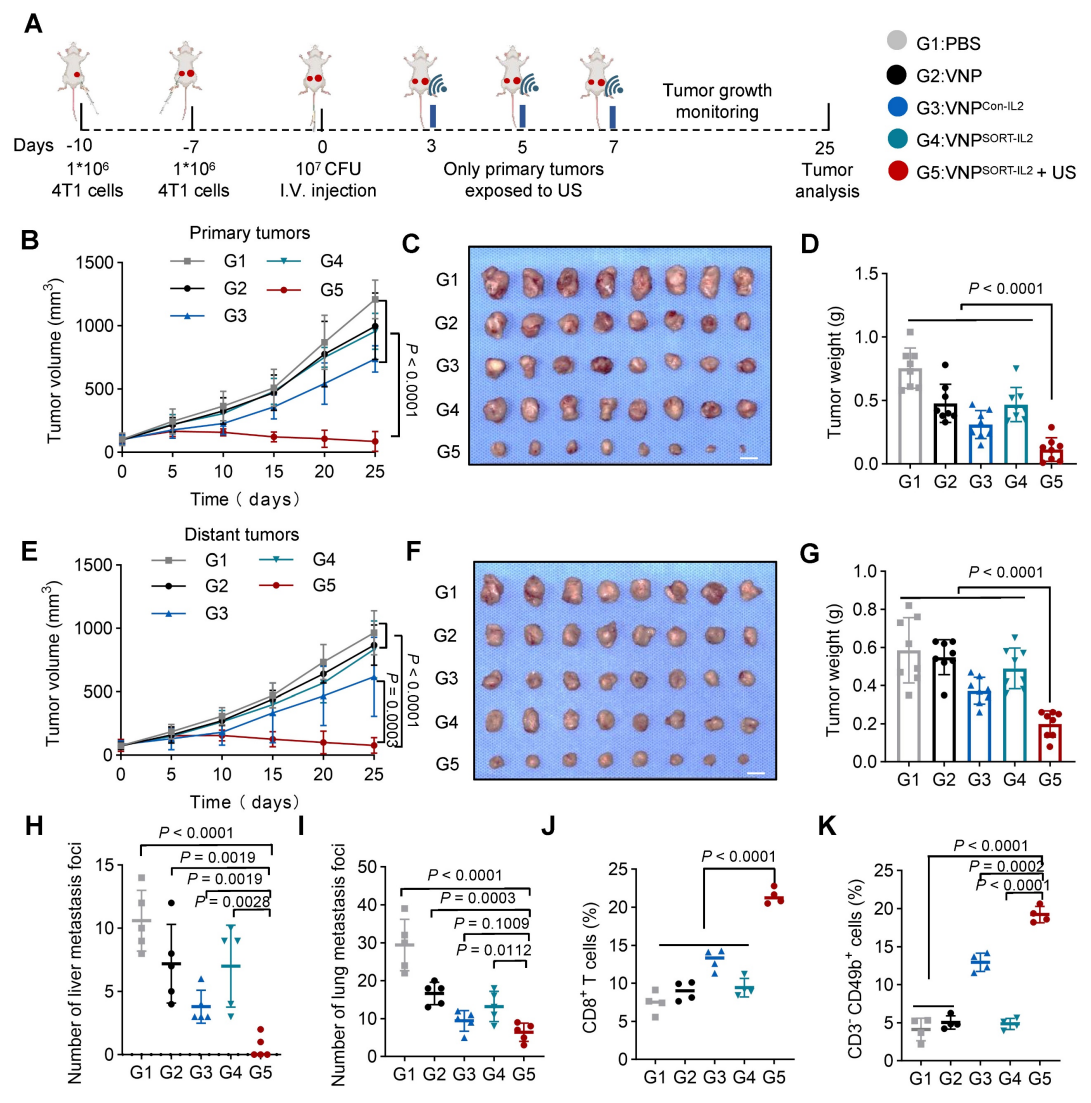
** VNP^SORT-IL2^ inhibits metastatic tumor growth. (A)** Schematic of the experimental procedure. Three days after tumor injection, BALB/c mice were additionally inoculated with 4T1 cells on the contralateral side. 4T1 tumor-bearing mice were then received intravenous injections of PBS, VNP, VNP^Con-IL2^, VNP^SORT-IL2^ or VNP^SORT-IL2^ +US at the indicated time. **(B)** Statistical analysis of the primary tumor growth with different treatments. **(C)** Excised primary tumors in indicated groups.** (D)** Primary tumor weights in different treatment groups. **(E)** Statistical analysis of the distant tumor growth with different treatments. **(F)** Excised distant tumors in indicated groups. **(G)** Distant tumor weights in different treatment groups. **(H)** The number of liver metastases. **(I)** The number of lung metastases. **(J-K)** Quantification of CD8^+^ T cells (CD3^+^CD8^+^). **(J)** and NK cells (CD3^-^CD49b^+^) **(K)** in distant tumor. Data are presented as mean ± SEM (n = 4-8 mice per group). *p* values were calculated by one-way ANOVA.

Given the robust antitumor immune activation elicited by VNP^SORT-IL2^ combined with US, we further established a distant/metastatic tumor model to evaluate systemic therapeutic efficacy. 4T1 cells were sequentially inoculated in the right flank (day 10) and left flank (day 7). One week later, when the right-flank tumors reached ~100 mm³, mice were administered PBS, VNP, VNP^Con-IL2^, or VNP^SORT-IL2^ *via* tail vein injection. US irradiation was applied to the right tumor on days 3, 5, and 7 post bacteria injection (Figure 8A). US irradiation selectively activated translation within the irradiated tumor, as evidenced by minimal fluorescence detected in the non-irradiated contralateral tumors (Figure S11). VNP^SORT-IL2^ + US treatment induced significant suppression of primary tumor growth (Figure 8B-D; Figure S12A). Distant tumors also exhibited marked growth inhibition in VNP^SORT-IL2^ + US group, compared to PBS, VNP, VNP^Con-IL2^, and VNP^SORT-IL2^ groups (Figure 8E-G; Figure S12B). Moreover, VNP^SORT-IL2^ + US effectively inhibited both liver and lung metastases (Figure 8H-I; Figure S13).

Flow cytometry analysis further revealed enhanced infiltration of CD8^+^ T cells (Figure 8J) and NK cell recruitment (Figure 8K; Figure S14) in the distant tumors of the VNP^SORT-IL2^ + US group. Collectively, these results demonstrated that VNP^SORT-IL2^ + US could induce a robust systemic antitumor immune response, thereby inhibiting the progression of distant tumors and metastatic dissemination.

### VNP^SORT-IL2^ and VNP^SORT-sPD1^ combination achieves robust therapeutic efficacy in A20 lymphoma murine model

Combination therapies are commonly utilized in tumor immunotherapy, with specific drug combinations customized to particular clinical scenarios 34-37. The SORT-mediated expression cassette is a modular system and ready for engineering of diverse therapeutic genes. We thus investigated the therapeutic efficacy of IL-2 combined with sPD1 in an A20 lymphoma tumor model. Consistent with observations in the 4T1 tumor model, biodistribution analysis on day 3 post-injection revealed selective bacterial colonization within A20 lymphoma tumors, exhibiting significantly greater accumulation in tumor tissue relative to normal organs (Figure S15).

**Figure 9:**
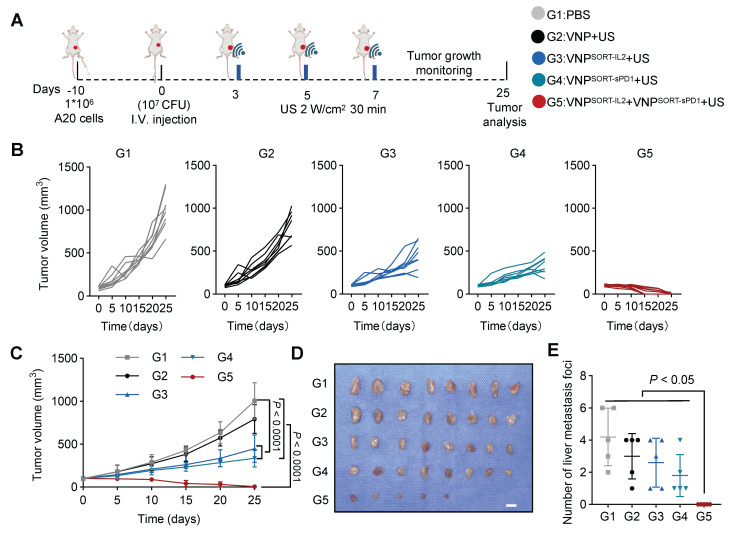
** Combination of VNP^SORT-IL2^ and VNP^SORT-sPD1^ inhibits A20 lymphoma. (A)** Schematic of the experimental procedure. BALB/c mice bearing A20 lymphoma tumors received intravenous injections of PBS, VNP, VNP^SORT-IL2^, VNP^SORT-sPD1^ or VNP^SORT-IL2^ and VNP^SORT-sPD1^, followed by US irradiation at indicated time. **(B)** Individual growth curves of A20 lymphoma tumors in different groups. **(C)** Statistical analysis of tumor growth in mice with different treatments. **(D)** Excised tumors from the mice. **(E)** Liver metastatic nodule numbers in mice with indicated treatments. Data are presented as mean ± SEM (n = 5-8 mice per group). *p* values were calculated by one-way ANOVA.

VNP^SORT-sPD1^ was constructed by inserting sPD1 into the SORT cassette (Figure S16A). *In vivo* experiments confirmed that US induction triggered sPD-1 secretion at the tumor site after injection of VNP^SORT-sPD1^ (Figure S16B-C). A20 cells inoculated mice were treated with PBS, VNP, VNP^SORT-IL2^, VNP^SORT-sPD1^, or a combination of VNP^SORT-IL2^ and VNP^SORT-sPD1^. US induction was performed on days 3, 5, and 7 post-injections (Figure 9A). Compared to monotherapy, the IL-2 and sPD-1 combination significantly enhanced anti-tumor effects (Figure 9B-C; Figure S17). Tumors were completely eliminated in 37.5% of mice (3/8) (Figure 9D), with no visible liver metastases detected (Figure 9E; Figure S18). These results demonstrated that the SORT platform could enable tumor-localized delivery of tailored therapeutic combinations.

## Discussion

In this study, we have engineered a SORT expression cassette to remotely regulate the therapeutic gene translation by brief US induction. In the SORT system, the RT was modified to harbor QRE, and thus could be opened by US irradiation and stabilized after QKI recognition. The therapeutic ORF following the RT could be translationally activated by US. With the engineered attenuated *Salmonella typhimurium* VNP20009 expressing mutated IL-2 or sPD-1 driven by SORT, US could spatiotemporally trigger production and secretion of IL-2 or sPD-1 in various syngeneic tumor mouse models, thereby achieving robust therapeutic efficacy while minimizing toxic effects.

US-activated gene circuits hold great promise for bacteria-based therapy, while continuous US exposure is typically required to induce enough therapeutic gene expression. Brief US irradiation that triggers durable gene expression clearly offers greater advantages, especially concerning the toxicity of US thermogenic effects. Prolonged US exposure may cause excessive local heating in normal tissues adjacent to tumors which not only causes side-effects but also dampens anti-tumor immunity 25. Compared to heat shock promoters, which typically require time to regulate transcription *via* host RNA polymerase/σ factors 38, RNA thermometer-based cassette enables faster responses. Compared to the canonical RNA thermometer, the current SORT system regulates durable gene expression *via* RNA and RNA-binding protein (RNA-RBP) interactions. In fact, hundreds of RBPs have been discovered and investigated over the years 39, and optimizing RNA-RBP pairs with higher thermos-sensitivity and controllability for therapeutic applications is worthy of exploration.

IL-2 was the first immunotherapy approved by the US Food and Drug Administration (FDA) in 1992 for the treatment of metastatic renal cell carcinoma 40. Nevertheless, systemic delivery of IL-2 for cancer therapy presents several significant challenges, including severe non-specific toxicities and the activation of regulatory T cells. To address these limitations, SORT-IL2 system represents an integration of recent advancements in ultrasound-responsive components, bacterial gene expression regulation, and IL-2 molecular engineering.

In the context of cancer immunotherapy, diverse therapeutic combinations are required for different disease stages and individual patients 41. On-demand development of precision therapies would significantly expand clinical applications. The SORT system is a modular platform that allows for easy replacement of the therapeutic genes. Notably, compared to co-expressing IL-2 and sPD-1 in a single bacterial cell, expressing them in separate bacteria has the following advantages. It prevents cross-gene expression interference, reduces bacterial metabolic stress to maintain colonization capacity, allows flexible adjustment of therapeutic ratios based on tumor characteristics, and enables more precise spatiotemporal control *via* US. Moreover, combination of bacteria armed with different therapeutics is easy in terms of clinical application. Rapid advancements in US technology now enable the development of intelligent wearable US patches 42-44. These devices could utilize flexible transducer arrays integrated with Bluetooth or Wi-Fi modules for remote parameter adjustment, making SORT based bacteria-US combination therapy more accessible, accurate, and applicable.

Overall, the proposed strategy combines the direct tumor-targeting capability of bacteria with the advantages of US in penetration depth and precision. This strategy holds considerable promise for the treatment of metastatic tumors where surgical removal is not feasible.

## Supplementary Material

Supplementary figures and tables.

## Abbreviations

TME: tumor microenvironment
HRE: hypoxia response element
Pnorv: promoter for NO detoxification gene norV
adiA: arginine decarboxylase
IPTG: isopropyl β-D-thiogalactoside
tTA: tetracycline-controlled transcriptional activator
rtTA: reverse tetracycline-controlled transcriptional activator
US: ultrasound
TSRs: temperature-sensitive repressors
PL-PR: leftward and rightward promoters
RTs: RNA thermometers
RBS: ribosome binding site
SORT: stabilized open RNA thermometer
QRE: QKI response element
ORF: open reading frame
sPD-1: soluble PD-1
RPMI: roswell park memorial institute
PBS: phosphate-buffered saline
qPCR: Quantitative PCR
H&E: hematoxylin and eosin
oRT: original RT
IFN-γ: interferon-γ
RNA-RBP: RNA and RNA-binding protein
FDA: food and drug administration
