# Supplementary material for: Ultrasound spatiotemporally enables prolonged therapeutic mRNA translation in engineered bacteria for enhanced cancer immunotherapy
Source: Theranostics. 2025 Aug 30;15(18):9742–56. doi: 10.7150/thno.120342 (PMC12486422; doi:10.7150/thno.120342)
Supplement: Supplementary file 1 — Supplementary figures and tables. [file thnov15p9742s1.pdf]

Supplementary Materials

Ultrasound spatiotemporally enables prolonged therapeutic mRNA translation in engineered bacteria for enhanced cancer immunotherapy

Zhaoyou Liu[1†], Lantian Wang[1†], Jieyuan An[1†], Tian Zhou[1], Mengying Wei[2], Guodong Yang[2], Pengying Wu[1✉], and Lijun Yuan[1✉]

Supporting Information Captions

Table S1. The plasmids used in this study.

Table S2. The ribosome binding sites used in this study.

Table S3. Primers used in this study.

Figure S1. Sequences and structures of the original RNA thermometer (oRT) and modified RT (mRT).

Figure S2. Effects of gene engineering on bacterial activity.

Figure S3. Optimization of the SORT expression system.

Figure S4. Setup of US treatment *in vitro*.

Figure S5. Gene expression pattern in VNP$^{Con\text{-}IL2}$ and VNP$^{SORT\text{-}IL2}$ treated with or without US treatment.

Figure S6. 4T1 breast tumor-targeting properties of VNP20009 bacteria.

Figure S7. Temperature dependent activation of the SORT system and effects of VNP20009 injection on body temperature.

Figure S8. Setup of US treatment *in vivo.*

Figure S9. IL-2 leakage from engineered bacteria in normal tissues.

Figure S10. Gating strategies of T cells and macrophages.

Figure S11. Bacterial gene expression in bilateral tumors.

Figure S12. Individual growth curves of 4T1 bilateral tumors.

Figure S13. Representative images of liver metastases in 4T1 tumor-bearing mice with different treatments.

Figure S14. Gating strategies of NK cells

Figure S15. Tumor targeting of VNP20009 in A20 tumors.

Figure S16. Construction and characterization of engineered bacteria for US-responsive sPD1 secretion.

Figure S17. Tumor weight of the different treatment groups.

Figure S18. Representative images of liver metastases in A20 tumor-bearing mice with different treatments.

Supplemental Tables

Table S1. The plasmids used in this study.

Plasmid	Sequence
J23100-RT-mCherry	TTGACGGCTAGCTCAGTCCTAGGTACAGTGCTAGCATGACTAAC<br>TTGCTTAATCTAAGGAGTTTATGACCATGGTGAGCAAGGGCGAG<br>GAGGATAACATGGCCATCATCAAGGAGTTCATGCGCTTCAAGGT<br>GCACATGGAGGGCTCCGTGAACGGCCACGAGTTCGAGATCGAG<br>GGCGAGGGCGAGGGCCGCCCCTACGAGGGCACCCAGACCGCCA<br>AGCTGAAGGTGACCAAGGGTGGCCCCCTGCCCTTCGCCTGGGAC<br>ATCCTGTCCCCTCAGTTCATGTACGGCTCCAAGGCCTACGTGAA<br>GCACCCCGCCGACATCCCCGACTACTTGAAGCTGTCCTTCCCCG<br>AGGGCTTCAAGTGGGAGCGCGTGATGAACTTCGAGGACGGCGG<br>CGTGGTGACCGTGACCCAGGACTCCTCCCTCCAGGACGGCGAGT<br>TCATCTACAAGGTGAAGCTGCGCGGCACCAACTTCCCCTCCGAC<br>GGCCCCGTAATGCAGAAGAAGACCATGGGCTGGGAGGCCTCCTC<br>CGAGCGGATGTACCCCGAGGACGGCGCCCTGAAGGGCGAGATC<br>AAGCAGAGGCTGAAGCTGAAGGACGGCGGCCACTACGACGCTG<br>AGGTCAAGACCACCTACAAGGCCAAGAAGCCCGTGCAGCTGCC<br>CGGCGCCTACAACGTCAACATCAAGTTGGACATCACCTCCCACA<br>ACGAGGACTACACCATCGTGGAACAGTACGAACGCGCCGAGGG<br>CCGCCACTCCACCGGCGGCATGGACGAGCTGTACAAGTAG
J23100- RT -mCherry-RBS-QKI	TTGACGGCTAGCTCAGTCCTAGGTACAGTGCTAGCATGACTAAC<br>TTGCTTAATCTAAGGAGTTTATGACCATGGTGAGCAAGGGCGAG<br>GAGGATAACATGGCCATCATCAAGGAGTTCATGCGCTTCAAGGT<br>GCACATGGAGGGCTCCGTGAACGGCCACGAGTTCGAGATCGAG<br>GGCGAGGGCGAGGGCCGCCCCTACGAGGGCACCCAGACCGCCA<br>AGCTGAAGGTGACCAAGGGTGGCCCCCTGCCCTTCGCCTGGGAC

	ATCCTGTCCCCTCAGTTCATGTACGGCTCCAAGGCCTACGTGAA GCACCCCGCCGACATCCCCGACTACTTGAAGCTGTCCTTCCCCG AGGGCTTCAAGTGGGAGCGCGTGATGAACTTCGAGGACGGCGG CGTGGTGACCGTGACCCAGGACTCCTCCCTCCAGGACGGCGAGT TCATCTACAAGGTGAAGCTGCGCGGCACCAACTTCCCCTCCGAC GGCCCCGTAATGCAGAAGAAGACCATGGGCTGGGAGGCCTCCTC CGAGCGGATGTACCCCGAGGACGGCGCCCTGAAGGGCGAGATC AAGCAGAGGCTGAAGCTGAAGGACGGCGGCCACTACGACGCTG AGGTCAAGACCACCTACAAGGCCAAGAAGCCCGTGCAGCTGCC CGGCGCCTACAACGTCAACATCAAGTTGGACATCACCTCCCACA ACGAGGACTACACCATCGTGGAACAGTACGAACGCGCCGAGGG CCGCCACTCCACCGGCGGCATGGACGAGCTGTACAAGTAGTAAG GAGGTATACATATGTATGTGCCTGTAAAAGAATACCCTGATTTT AATTTTGTTGGGAGAATCCTTGGACCTAGAGGACTTACAGCTAA ACAACTTGAAGCAGAAACGGGATGTAAAATAATGGTCCGAGGC AAAGGCTCAATGAGGGATAAAAAGAAGGAGGAGCAAAATAGA GGCAAGCCCAATTGGGAGCATCTAAATGAAGACTTACATGTACT AATCACTGTGGAAGATGCTCAGAACAGAGCAGAAATCAAGCTG AAGAGAGCGGTTGAAGAAGTGAAGAAGTTACTGGTACCTGCGG CTGAAGGTGAAGACAGCCTGAAGAAGATGCAGCTGATGGAGCT TGCAATTCTGAATGGCACCTACAGAGACGCCAACATTAAATCAC CAGCCTGA
J23100-RBS-QKI	TTGACGGCTAGCTCAGTCCTAGGTACAGTGCTAGCTCACACAGG ACTATACATATGTATGTGCCTGTAAAAGAATACCCTGATTTTAAT TTTGTTGGGAGAATCCTTGGACCTAGAGGACTTACAGCTAAACA ACTTGAAGCAGAAACGGGATGTAAAATAATGGTCCGAGGCAAA GGCTCAATGAGGGATAAAAAGAAGGAGGAGCAAAATAGAGGCA AGCCCAATTGGGAGCATCTAAATGAAGACTTACATGTACTAATC ACTGTGGAAGATGCTCAGAACAGAGCAGAAATCAAGCTGAAGA

	GAGCGGTTGAAGAAGTGAAGAAGTTACTGGTACCTGCGGCTGA AGGTGAAGACAGCCTGAAGAAGATGCAGCTGATGGAGCTTGCA ATTCTGAATGGCACCTACAGAGACGCCAACATTAAATCACCAGC CTGA
J23100- RBS-mCherry	TTGACGGCTAGCTCAGTCCTAGGTACAGTGCTAGCTCACACAGG ACTATACATATGGTGAGCAAGGGCGAGGAGGATAACATGGCCA TCATCAAGGAGTTCATGCGCTTCAAGGTGCACATGGAGGGCTCC GTGAACGGCCACGAGTTCGAGATCGAGGGCGAGGGCGAGGGCC GCCCCTACGAGGGCACCCAGACCGCCAAGCTGAAGGTGACCAA GGGTGGCCCCCTGCCCTTCGCCTGGGACATCCTGTCCCCTCAGTT CATGTACGGCTCCAAGGCCTACGTGAAGCACCCCGCCGACATCC CCGACTACTTGAAGCTGTCCTTCCCCGAGGGCTTCAAGTGGGAG CGCGTGATGAACTTCGAGGACGGCGGCGTGGTGACCGTGACCCA GGACTCCTCCCTCCAGGACGGCGAGTTCATCTACAAGGTGAAGC TGCGCGGCACCAACTTCCCCTCCGACGGCCCCGTAATGCAGAAG AAGACCATGGGCTGGGAGGCCTCCTCCGAGCGGATGTACCCCGA GGACGGCGCCCTGAAGGGCGAGATCAAGCAGAGGCTGAAGCTG AAGGACGGCGGCCACTACGACGCTGAGGTCAAGACCACCTACA AGGCCAAGAAGCCCGTGCAGCTGCCCGGCGCCTACAACGTCAAC ATCAAGTTGGACATCACCTCCCACAACGAGGACTACACCATCGT GGAACAGTACGAACGCGCCGAGGGCCGCCACTCCACCGGCGGC ATGGACGAGCTGTACAAGTAG
J23100- RT - YopE-IL2-RBS-QKI	TTGACGGCTAGCTCAGTCCTAGGTACAGTGCTAGCATGACTAAC TTGCTTAATCTAAGGAGTTTATGACCATGAAAATATCATCATTTA TTTCTACATCACTGCCCCTGCCGACAGCACCTACTTCAAGTTCTA CAAAGAAAACACAGCTACAACTGGAGCATTTACTGCTGGATTTA CAGATGATTTTGAATGGAATTAATAATTACAAGAATCCCAAACT CACCAGGATGCTCACATTTAAGTTTTACATGCCCAAGAAGGCCA CAGAACTGAAACATCTTCAGTGTCTAGAAGAAGAACTCAAACCT

	CTGGAGGAAGTGCTAAATTTAGCTCAAAGCAAAAACTTTCACTTTGATCCCAGGGACGTAGTAAGCAATATCAACGTATTTGTTCTGGAACTAAAGGGATCTGAAACAACATTCATGTGTGAATATGCTGATGAGACAGCAACCATTGTAGAATTTCTGAACAGATGGATTACCTTTTGTCAAAGCATCATCTCAACACTGACTTGATCACACAGGACTATACATATGTATGTGCCTGTAAAAGAATACCCTGATTTTAATTTTGTTGGGAGAATCCTTGGACCTAGAGGACTTACAGCTAAACAACTTGAAGCAGAAACGGGATGTAAAATAATGGTCCGAGGCAAAGGCTCAATGAGGGATAAAAAGAAGGAGGAGCAAAATAGAGGCAAGCCCAATTGGGAGCATCTAAATGAAGACTTACATGTACTAATCACTGTGGAAGATGCTCAGAACAGAGCAGAAATCAAGCTGAAGAGAGCGGTTGAAGAAGTGAAGAAGTTACTGGTACCTGCGGCTGAAGGTGAAGACAGCCTGAAGAAGATGCAGCTGATGGAGCTTGCAATTCTGAATGGCACCTACAGAGACGCCAACATTAAATCACCAGCCCATCATCACCATCACCATTGA
J23100-RBS-YopE-IL2	TTGACGGCTAGCTCAGTCCTAGGTACAGTGCTAGCTCACACAGGACTATACATATGAAAATATCATCATTTATTTCTACATCACTGCCCCTGCCGACAGCACCTACTTCAAGTTCTACAAAGAAAACACAGCTACAACTGGAGCATTTACTGCTGGATTTACAGATGATTTTGAATGGAATTAATAATTACAAGAATCCCAAACTCACCAGGATGCTCACATTTAAGTTTTACATGCCCAAGAAGGCCACAGAACTGAAACATCTTCAGTGTCTAGAAGAAGAACTCAAACCTCTGGAGGAAGTGCTAAATTTAGCTCAAAGCAAAAACTTTCACTTTGATCCCAGGGACGTAGTAAGCAATATCAACGTATTTGTTCTGGAACTAAAGGGATCTGAAACAACATTCATGTGTGAATATGCTGATGAGACAGCAACCATTGTAGAATTTCTGAACAGATGGATTACCTTTTGTCAAAGCATCATCTCAACACTGACTTGA
J23100- RT -YopE-sPD1-	TTGACGGCTAGCTCAGTCCTAGGTACAGTGCTAGCATGACTAACTTGCTTAATCTAAGGAGTTTATGACCATGAAAATATCATCATTTA

RBS-QKI	TTTCTACATCACTGCCCCTGCCGACACTAGAGGTCCCCAATGGG CCCTGGAGGTCCCTCACCTTCTACCCAGCCTGGCTCACAGTGTCA GAGGGAGCAAATGCCACCTTCACCTGCAGCTTGTCCAACTGGTC GGAGGATCTTATGCTGAACTGGAACCGCCTGAGTCCCAGCAACC AGACTGAAAAACAGGCCGCCTTCTGTAATGGTTTGAGCCAACCC GTCCAGGATGCCCGCTTCCAGATCATACAGCTGCCCAACAGGCA TGACTTCCACATGAACATCCTTGACACACGGCGCAATGACAGTG GCATCTACCTCTGTGGGGCCATCTCCCTGCACCCCAAGGCAAAA ATCGAGGAGAGCCCTGGAGCAGAGCTCGTGGTAACAGAGAGAA TCCTGGAGACCTCAACAAGATATCCCAGCCCCTCGCCCAAACCA GAAGGCCGGTTTCAAGGCATGTGATCACACAGGACTATACATAT GTATGTGCCTGTAAAAGAATACCCTGATTTTAATTTTGTTGGGAG AATCCTTGGACCTAGAGGACTTACAGCTAAACAACTTGAAGCAG AAACGGGATGTAAAATAATGGTCCGAGGCAAAGGCTCAATGAG GGATAAAAAGAAGGAGGAGCAAAATAGAGGCAAGCCCAATTGG GAGCATCTAAATGAAGACTTACATGTACTAATCACTGTGGAAGA TGCTCAGAACAGAGCAGAAATCAAGCTGAAGAGAGCGGTTGAA GAAGTGAAGAAGTTACTGGTACCTGCGGCTGAAGGTGAAGACA GCCTGAAGAAGATGCAGCTGATGGAGCTTGCAATTCTGAATGGC ACCTACAGAGACGCCAACATTAAATCACCAGCCCATCATCACCA TCACCATTGA

Green shading represents the spacer sequences between RBS and ATG. Blue shading represents the sequences are mutated corresponding to the wildtype.

Table S2. The ribosome binding sites used in this study.

Candidate	Sequence
RBS1	TAAGGAGG
RBS2	TCACACAGGAC
RBS3	AAAGAGGAGAAA
RBS4	TCACACAGGAAACC

Table S3. Primers used in this study.

Gene	Forward primer (5'-3')	Reverse primer (5'-3')
IL-2	GCACCTACTTCAAGTTCTACAAAG	GTCAGTGTTGAGATGATGCTTT
16S rRNA	GTGCCAGCAGCCGCGGTAA	GGACTACCAGGGTATCTAAT

Supplemental figures

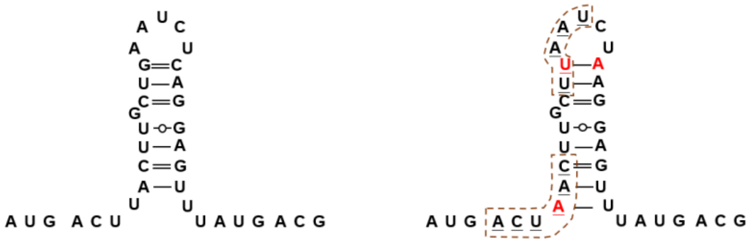

oRT　　　　mRT

Figure S1. Sequences and structures of the original RNA thermometer (oRT) and modified RT (mRT). The oRT is the natural *ibpA* RNA thermometer from *Pseudomonas putida.* The mutated sequence is highlighted in red, and the resultant QKI response elements are highlighted by the brown dashed box.

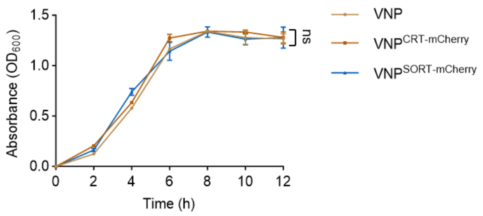

Figure S2. Effects of gene engineering on bacterial activity. The growth curves of VNP, VNP$^{CRT\text{-}mCherry}$ and VNP$^{SORT\text{-}mCherry}$ in LB medium at 37 °C were documented. The OD_{600} values were measured at 2-hour intervals using a microplate reader. Data are presented as mean ± SEM (n = 3 independent experiments). *p* values were calculated by one-way ANOVA.

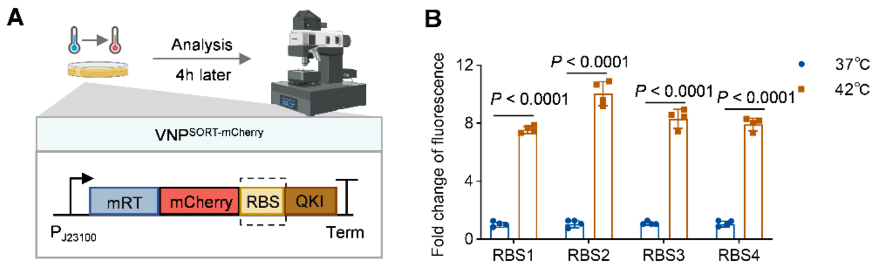

Figure S3. Optimization of the SORT expression system. (A) Schematic illustration of ribosome binding site (RBS) screen workflow. **(B)** Fold change of fluorescence of VNP$^{\text{SORT-mCherry}}$ containing different RBSs at 37 °C and 42 °C. Data are presented as mean ± SEM (n = 4 independent experiments). *p* values were calculated by Student's t-test.

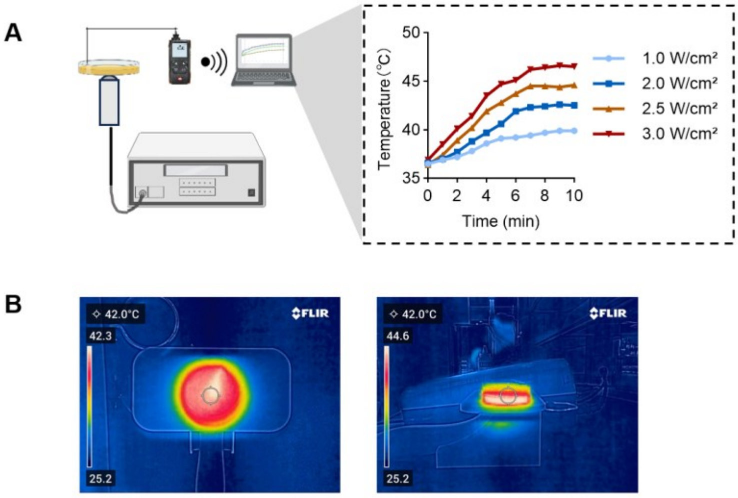

Figure S4. Setup of US treatment *in vitro*. (A) Schematic diagram for real-time detection of temperature increases in bacteria solution (left) and quantification of the temperature (right) irradiated with the different ultrasonic parameters. (B) Representative top-view and side-view infrared thermography demonstrating that ultrasound irradiation maintained the bacterial solution temperature at 42 °C.

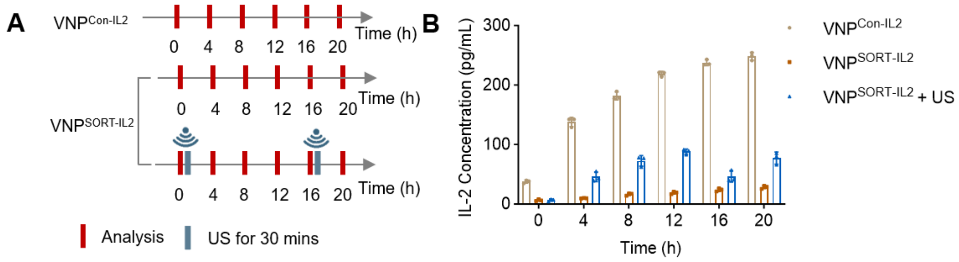

Figure S5. Gene expression pattern in VNP$^{Con\text{-}IL2}$ and VNP$^{SORT\text{-}IL2}$ treated with or without US treatment. (A) Schematic of the experimental procedure. (B) Quantitative analysis of IL-2 protein content in VNP$^{SORT\text{-}IL2}$ and VNP$^{Con\text{-}IL2}$ culture medium at different times.

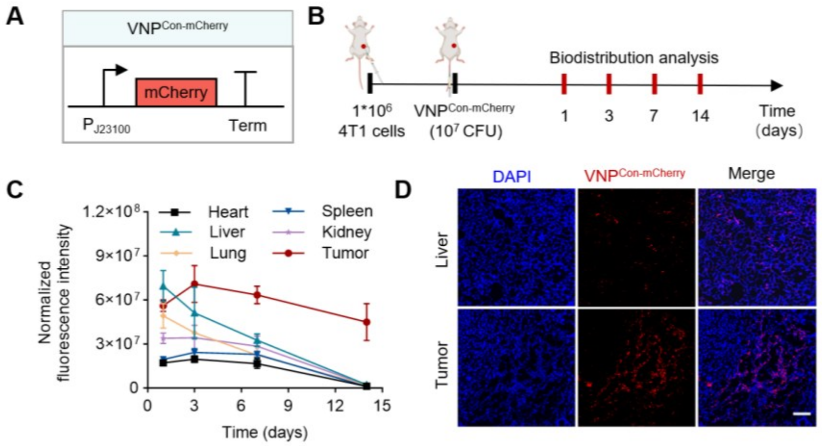

Figure S6. 4T1 breast tumor-targeting properties of VNP20009 bacteria. (A) Schematic representation of Con-mCherry plasmid. (B) Experimental procedure to verify the tumor-targeting property of the bacteria in 4T1 tumor-bearing mice. (C) Quantification of bacterial colonization in major organs and tumors collected at days 1, 3, 7, and 14. Data are presented as mean ± SEM (n = 3 independent experiments). (D) Representative fluorescence microscopy images showing $VNP^{Con\text{-}mCherry}$ localization in tumors and livers of tumor-bearing mice on day 7. Cells nuclei were stained with DAPI. scale bar = 100 μm.

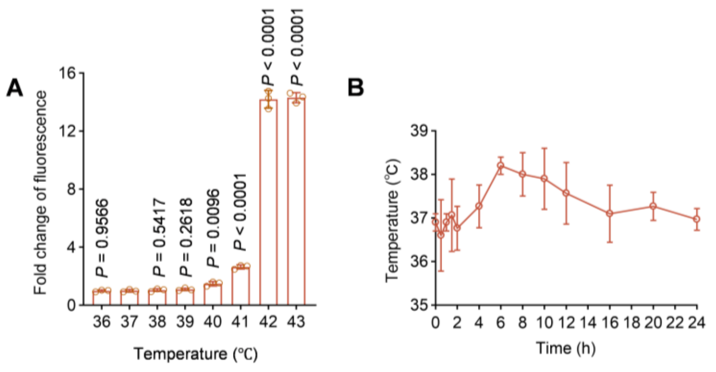

Figure S7. Temperature dependent activation of the SORT system and effects of VNP20009 injection on body temperature. (A) Fold change of fluorescence intensity in VNP$^{SORT\text{-}mCherry}$ at temperatures ranging from 36 to 43 °C. (B) Body temperature curves of mice within 24 hours after injection of VNP20009. Data are presented as mean ± SEM (n = 3 independent experiments). *p* values vs 37 °C were calculated by one-way ANOVA.

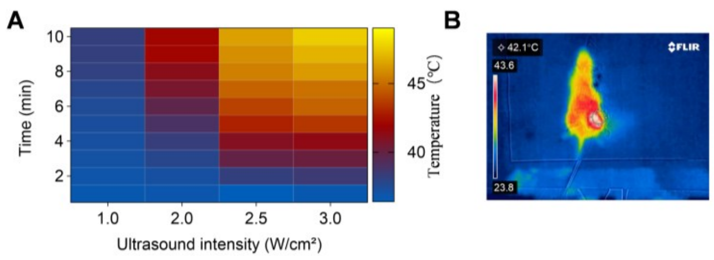

Figure S8. Setup of US treatment *in vivo*. (A) Heatmap visualization of the correlation between the temperature of tumor and the ultrasonic parameters tested. (B) Representative infrared thermography showing that focused ultrasound (2.0 W/cm^2, 30% duty cycle) maintained the temperature at approximately 42 °C at the tumor site.

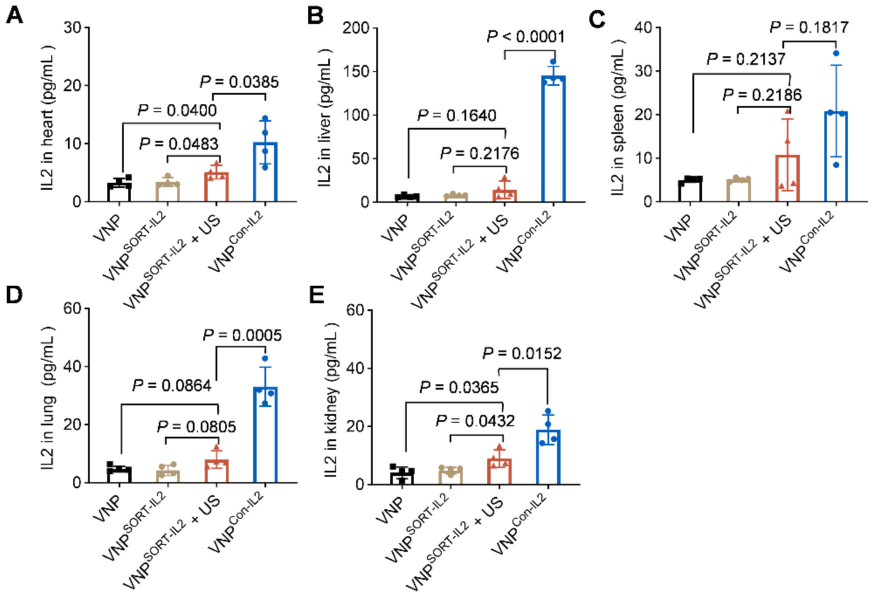

Figure S9. IL-2 leakage from engineered bacteria in normal tissues. (A-E) On day 3 after intravenous injection of bacteria in tumor-bearing mice, ultrasound induction was performed in the designated groups. Mice were euthanized, and the heart (A), liver (B), spleen (C), lung (D), and kidney (E) were harvested for homogenization 4 h later. IL-2 levels in normal tissues were then quantified by ELISA. Data are presented as mean ± SEM (n = 4 mice per group). *p* values were calculated by one-way ANOVA.

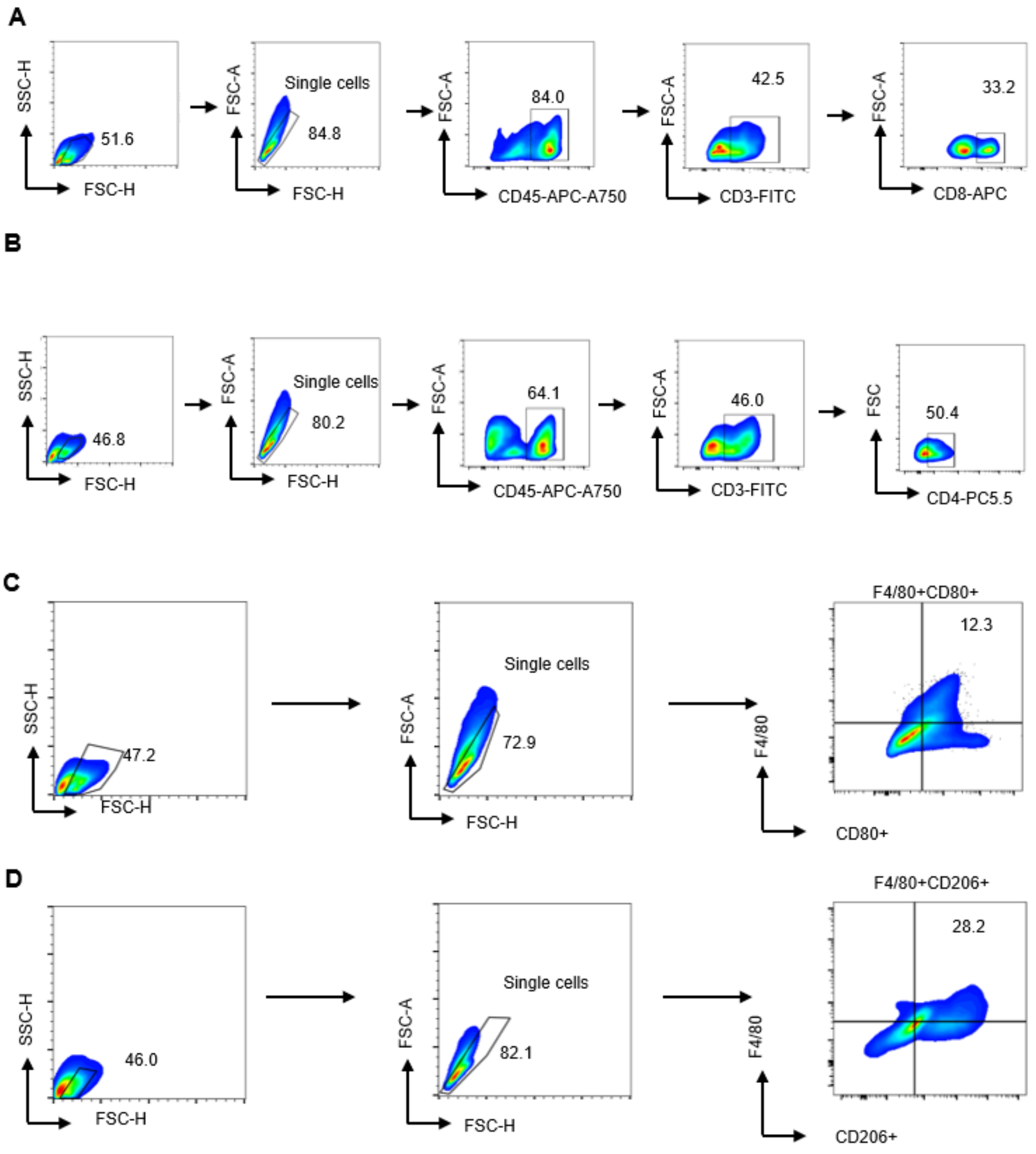

Figure S10. Gating strategies of T cells and macrophages. (A-B) Representative flow cytometry gating strategies for $CD45^+$ $CD3^+$ $CD8^+$ (A) and $CD45^+$ $CD3^+$ $CD4^+$ T cells (B). (C-D) Representative flow cytometry gating strategies for $F4/80^+$ $CD80^+$ (C) and $F4/80^+$ $CD206^+$ macrophage (D).

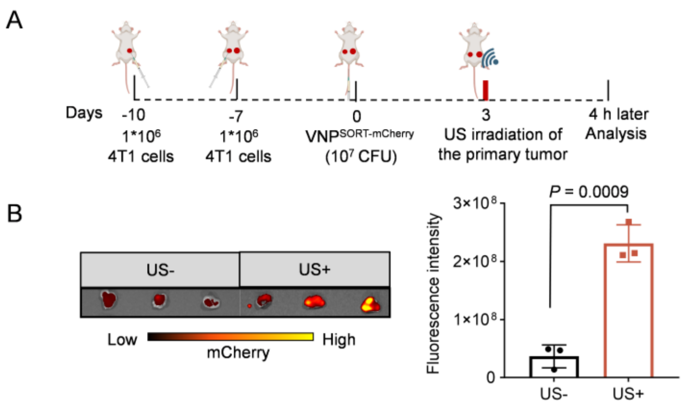

Figure S11. Bacterial gene expression in bilateral tumors. (A) Experimental procedure to verify bacterial gene expression in tumors on both sides either with or without ultrasound irradiation. (B) *Ex vivo* fluorescence imaging showing mCherry expression in bilateral tumors either with or without ultrasound irradiation. Data are presented as mean ± SEM (n = 3 independent experiments). p values were calculated by Student's t-test.

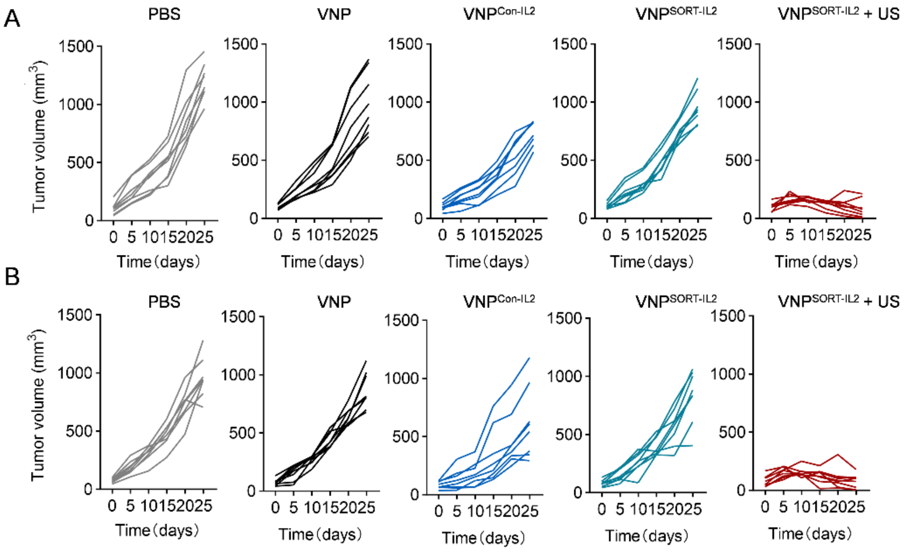

Figure S12. Individual growth curves of 4T1 bilateral tumors. (A) Individual growth curves of primary tumors in different treatment groups, including PBS, VNP, VNP$^{Con\text{-}IL2}$, VNP$^{SORT\text{-}IL2}$, VNP$^{SORT\text{-}IL2}$ +US. (B) Individual growth curves of distant tumors in different treatment groups, including PBS, VNP, VNP$^{Con\text{-}IL2}$, VNP$^{SORT\text{-}IL2}$, VNP$^{SORT\text{-}IL2}$ + US. n = 8 mice per group.

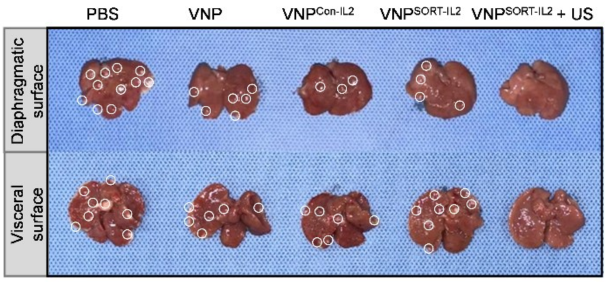

Figure S13. Representative images of liver metastases in 4T1 tumor-bearing mice with different treatments. On day 25 post-bacterial injection, mice from different treatment groups were euthanized. Livers were harvested, rinsed with PBS, and metastases were quantified on both the visceral and diaphragmatic surfaces of the liver. Metastases were marked with white circles.

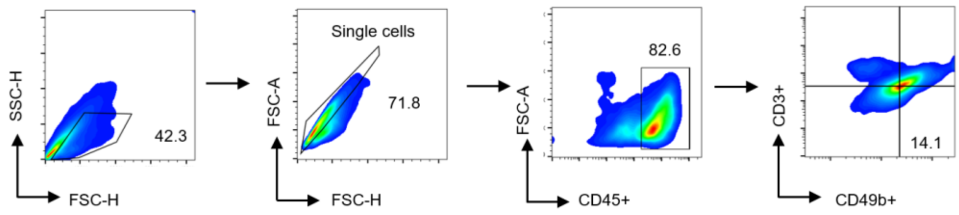

Figure S14. Gating strategies of NK cells. Representative flow cytometry gating strategies for NK cells ($CD45^{+}$ $CD3^{-}$ $CD49b^{+}$).

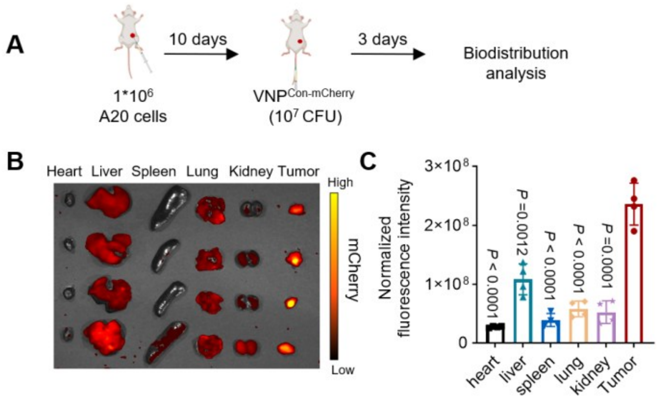

Figure S15. Tumor targeting of VNP20009 in A20 tumors. (A) Experimental procedure to verify the A20 tumor-targeting property of the bacteria at day 3. (B) Representative *ex vivo* fluorescent images of various organs and tumor tissues in mice, captured at day 3 following tail vein injection of VNP$^{Con\text{-}mCherry}$. (C) Quantification of bacterial colonization in major organs and tumors collected from tumor-bearing mice. Data are presented as mean ± SEM (n = 4 mice per group). p values vs tumor were calculated by one-way ANOVA.

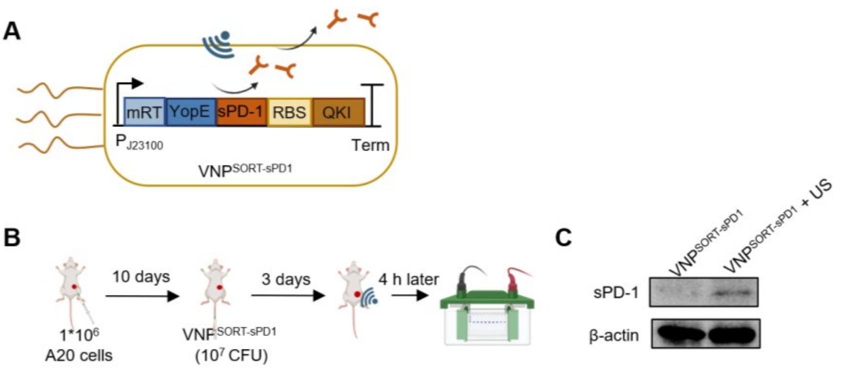

Figure S16. Construction and characterization of engineered bacteria for US-responsive sPD1 secretion. (A) VNP$^{SORT\text{-}sPD1}$ was constructed by replacing the IL-2 gene with sPD1. (B) Experimental procedures to verify whether ultrasound successfully triggered sPD1 secretion in the engineered bacteria at the tumor site. (C) Western blotting showed that ultrasound successfully triggered sPD1 secretion.

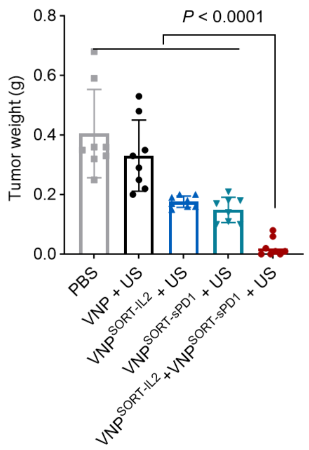

Figure S17. Tumor weight of the different treatment groups. On day 25 post-bacterial injection, mice from different treatment groups were euthanized and tumors were excised and weighed. Data are presented as mean ± SEM (n = 8 mice per group). *p* values were calculated by one-way ANOVA.

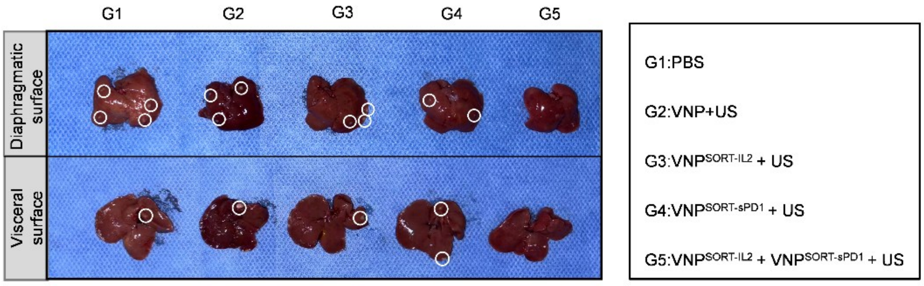

Figure S18. Representative images of liver metastases in A20 tumor-bearing mice with different treatments. On day 25 post-bacterial injection, mice from different treatment groups were euthanized. Livers were harvested, rinsed with PBS, and metastases were quantified on both the visceral and diaphragmatic surfaces of the liver. Metastases were marked with white circles.
